# Glucocorticoids modulate multidrug resistance transporters in the first trimester human placenta

**DOI:** 10.1111/jcmm.13646

**Published:** 2018-04-24

**Authors:** Phetcharawan Lye, Enrrico Bloise, Lubna Nadeem, William Gibb, Stephen J. Lye, Stephen G. Matthews

**Affiliations:** ^1^ Department of Physiology University of Toronto Toronto ON Canada; ^2^ Department of Morphology Federal University of Minas Gerais Belo Horizonte Brazil; ^3^ Lunenfeld‐Tanenbaum Research Institute Mount Sinai Hospital Toronto ON Canada; ^4^ Department of Obstetrics & Gynaecology University of Ottawa Ottawa ON Canada; ^5^ Department of Cellular & Molecular Medicine University of Ottawa Ottawa ON Canada; ^6^ Department of Obstetrics & Gynaecology University of Toronto Toronto ON Canada; ^7^ Department of Medicine Faculty of Medicine University of Toronto Toronto ON Canada

**Keywords:** BeWo, breast cancer related protein (BCRP), dexamethasone, first trimester placenta, glucocorticoids, P‐glycoprotein (P‐gp)

## Abstract

The placental multidrug transporters, P‐glycoprotein (P‐gp, encoded by *ABCB1*) and breast cancer resistance protein (BCRP,*ABCG2*) protect the foetus from exposure to maternally derived glucocorticoids, toxins and xenobiotics. During pregnancy, maternal glucocorticoid levels can be elevated by stress or exogenous administration. We hypothesized that glucocorticoids modulate the expression of *ABCB1*/P‐gp and *ABCG2*/BCRP in the first trimester human placenta. Our objective was to examine whether dexamethasone (DEX) or cortisol modulate first trimester placental expression of multidrug transporters and determine whether cytotrophoblasts or the syncytiotrophoblast are/is responsible for mediating these effects. Three models were examined: (i) an *ex‐vivo* model of placental villous explants (7‐10 weeks), (ii) a model of isolated first trimester syncytiotrophoblast and cytotrophoblast cells and (iii) the BeWo immortalized trophoblast cell line model. These cells/tissues were treated with DEX or cortisol for 24 hour to 72 hour. In first trimester placental explants, DEX (48 hour) increased *ABCB1* (*P* < .001) and *ABCG2* (*P* < .05) mRNA levels, whereas cortisol (48 hour) only increased *ABCB1* mRNA levels (*P* < .01). Dexamethasone (*P* < .05) and cortisol (*P* < .01) increased BCRP but did not affect P‐gp protein levels. Breast cancer resistance protein expression was primarily confined to syncytiotrophoblasts. BeWo cells, when syncytialized with forskolin, increased expression of BCRP protein, and this was further augmented by DEX (*P* < .05). Our data suggest that the protective barrier provided by BCRP increases as cytotrophoblasts fuse to form the syncytiotrophoblast. Increase in glucocorticoid levels during the first trimester may reduce embryo/foetal exposure to clinically relevant BCRP substrates, because of an increase in placental BCRP.

## 1 INTRODUCTION

The placenta supplies nutrition to the growing foetus while simultaneously forming a barrier which protects the foetus from exposure to hormones, drugs and toxins (including glucocorticoids, organophosphate pesticides and endocrine disruptors) present in the maternal circulation.^1^, ^2^, ^3^ This barrier function is supported by the ABC family of efflux transporters.^4^ In this regard, the multidrug resistance transporters, P‐glycoprotein (P‐gp; encoded by the *ABCB1* gene) and breast cancer resistance protein (BCRP [breast cancer resistance protein]; encoded by the *ABCG2* gene) are primarily localized to the apical membrane of the syncytiotrophoblast. They efflux substrates from within the syncytiotrophoblast back into the maternal circulation hence preventing factors present in the maternal blood from entering the foetal compartment.^3^

In normal gestation, placental P‐gp levels are high during first trimester and decline with advancing gestation, whereas placental BCRP levels increase towards term.^3^, ^5^, ^6^ In addition, altered expression of these transporters has been identified in reproductive disorders and in pathological pregnancies.^4^ Therefore, the biodistribution of their substrates in the foetal compartment is gestational age dependent and may be altered in obstetric pathologies.

Maternal levels of glucocorticoids can be increased by stress in pregnancy and by administration of synthetic glucocorticoids (sGC) to pregnant women. In the early stages of pregnancy, sGC may be used in conjunction with other drugs as part of immunomodulatory treatments to improve the outcomes of *in vitro* fertilization^7^ or given in early gestation to prevent virilization in female foetuses, where there is a risk of congenital adrenal hyperplasia (CAH).^8^, ^9^, ^10^ The effects of sGC, such as Dexamethasone (DEX), are mediated via activation of the glucocorticoid receptor (GR). In contrast, endogenous glucocorticoids (eg, cortisol) act through the GR and the mineralocorticoid receptor (MR). Once activated, these receptors act as transcription factors and bind to the glucocorticoid response element (GRE) in the regulatory region of their target genes.^11^, ^12^

We have previously shown that sGC modulate expression of P‐gp in the murine placenta. Expression of placental *Abcb1*a mRNA and P‐gp protein was increased, whereas expression of placental *Abcg2* mRNA was decreased and BCRP function inhibited in the mouse treated with sGC.^13^, ^14^, ^15^ In the guinea pig, corticosteroid treatment induced P‐gp function in the developing blood‐brain barrier,^13^ while betamethasone decreased placental *Abcb1* mRNA and P‐gp protein expression in late gestation,^16^ demonstrating tissue‐specific regulation. Furthermore, human third trimester preterm labor (PTL)‐threatened pregnancies exposed to antenatal betamethasone therapy did not exhibit deranged *ABCB1* and P‐gp expression.^17^ However, increased maternal distress was directly associated with altered term placental expression of *ABCB1* and *ABCG2* mRNA levels,^18^ suggesting that glucocorticoids have the potential to modulate the expression of multidrug resistance transporters in the third trimester placenta in certain conditions.

While evidence points to a regulatory action of glucocorticoids on placental multidrug resistance in the later stages of pregnancy, very little is known about the effect of glucocorticoids regulating P‐gp/*ABCB1* and BCRP/*ABCG2* in the human first trimester placenta. We hypothesized that glucocorticoids modulate the expression of P‐gp and BCRP in the first trimester human placenta and that these effects are trophoblast lineage‐specific. Therefore, in this study, we examined whether DEX or cortisol altered the placental expression of these multidrug transporters in the first trimester placenta. Further, we determined how trophoblast fusion into syncytium modifies transporter expression and if this is affected by subsequent glucocorticoid exposure.

## 2 MATERIALS AND METHODS

### 2.1 Tissue collection

First trimester tissues were collected at 7‐10 weeks of pregnancy by the Research Centre for Women's and Infants’ Health Bio Bank program at Mount Sinai Hospital after informed consent and in adherence with the policies of Mount Sinai Hospital and the University of Toronto Research Ethic Boards.

### 2.2 Placental villous explant culture

Placental villous explants were cultured as described previously,^3^, ^19^ with minor modifications. Briefly, placental specimens were placed into phosphate‐buffered saline (1%; PBS) without Ca^+^ and Mg^+^ and transported to the laboratory. Tissues were dissected into villous clusters of approximately 15‐30 mg, and 3 villous explants were cultured per well in 12‐well plates that contained Dulbecco's modified Eagle's medium/F12, normocin antibiotic (Invivogen, San Diego, CA), and 1X insulin, transferrin and selenium‐A (Invitrogen, Grand Island, NY) that was previously equilibrated at 8% O_2_ (5% CO_2_, 37°C) for 24 hour. Explants were cultured for 24 hour and then randomly assigned into treatment groups. Explants were treated with DEX or cortisol (10^−8^ or 10^−6 ^mol/L; Sigma‐Aldrich, St. Louis, MO), or vehicle for either 24 hour or 48 hour. Explants were then collected and stored at −80°C for total RNA and protein extraction. The culture media was collected to measure lactate dehydrogenase (LDH) in order to assess tissue viability during culture (Roche Applied Science, Indianapolis, IN) as previously described.^3^, ^19^

### 2.3 BeWo cell culture

The human choriocarcinoma‐derived cell line BeWo was obtained from ATCC (Burlington, ON, Canada) and cultured as described previously.^20^, ^21^ Briefly, cells were cultured in DMEM/F12 medium supplemented with 10% charcoal‐stripped foetal bovine serum (WISENT Inc. Quebec, Ca), 100 IU/mL of penicillin and 100 μg/mL of streptomycin at 8% O_2_ (5% CO_2_, 37^ο^C) (Invitrogen Canada Inc., Burlington, ON, Canada). Cells were seeded (4 × 10^4^ per well, respectively) in 6‐well plates and cultured for 24 hour at 8% O_2_ (5% CO_2_, 37°C). Syncytialization of BeWo cells was induced by treatment with forskolin (25 μmol/L; Sigma‐Aldrich) for 72 hour and subsequently these cells were treated with DEX (10^−6 ^mol/L) or vehicle for a further 72 hour at 2% O_2_ (5% CO_2_, 37°C). Non‐syncytialized BeWo cells (control) were treated with DEX (10^−6^ mol/L) or vehicle for 72 hour at 2% O_2_. After treatments, cells were then collected and stored at −80°C for total RNA and protein extraction.

### 2.4 First trimester human primary cytotrophoblast and syncytiotrophoblast cell isolation

Cytotrophoblast and syncytiotrophoblast cells were isolated from 7‐ to 10‐week placentae as previously described^22^ with some modifications. Briefly, placental villous explants were dissected and digested (20 minutes) in Trypsin Digestion Cocktail (1M Hepes (5 mL; Sigma‐Aldrich), DNase I (20 mg; Sigma‐Aldrich), 0.85 mL of 1 mol/L MgSO_4_, Trypsin (5%, 10 mL), fungizone (2 mL), gentamicin (100 μg/mL; all from Invitrogen) and Hank's buffered salt solution (200 mL, HBSS) without Mg^2+^ and Ca^2+^, to remove the syncytiotrophoblast layer. Subsequently, the pellet of syncytial cells was washed with HBSS, centrifuged and the supernatant removed. Cells were collected for total protein extraction. The remaining cells were exposed to three 20‐minutes digestions for the collection of subsequent layers containing primary cytotrophoblast cells. Cells were collected and after the third digestion were washed with HBSS. Supernatant was removed, and protein extraction was performed.

### 2.5 Immunohistochemistry

After culture with DEX or cortisol, explants were processed for immunohistochemical analysis as previously described.^3^, ^19^ Slides were incubated (overnight at 4°C) with primary antibodies: anti‐mouse BCRP (1:200, BXP‐21, Millipore, Billerica, MA), and anti‐mouse cytokeratin 7 (CK7, 1:500, Dako). Mouse or rabbit IgG1 (Dako) was added instead of primary antibody in controls. After incubation, slides were washed and incubated with the corresponding biotinylated secondary antibody (1:300, 1 hour, Dako). Sections were then washed in PBS (3x for 10′ each time) and incubated with streptavidin‐HRP (30 minutes; Dako). Chromogenic detection of horseradish peroxidase (HRP) activity was achieved by 3,3′‐diaminobenzidine (DAB) reagent (DAB peroxidase substrate kit, Dako). Slides were counterstained with haematoxylin, dehydrated in ascending grades of ethanol and cover slipped. Slides were visualized with an Olympus BX61 upright, motorized microscope with an Olympus DP72 digital camera (Olympus, Tokyo, Japan).

### 2.6 qPCR

Total RNA was isolated from the first trimester explants and placental cell line with RNeasy Plus Universal Mini Kit (Qiagen, Toronto, ON, Canada), as previously described.^3^, ^19^ RNA concentration and purity were assessed with NanoDrop1000 Spectrophotometer (Thermo Scientific, Wilmington, DE) and Experion RNA StdSens Analysis Kit (Bio‐Rad, Mississauga, ON, Canada), respectively. RNA was reverse‐transcribed into cDNA using the iScript Reverse Transcription Supermix (Bio‐Rad). mRNA levels of the *ABCB1*,* ABCG2*,* GR* and *MKP‐1/DUSP1* (MAP kinase phosphatase‐1) genes were measured by qPCR using SYBR Green reagent (Sigma‐Aldrich) and the CFX 380 Real‐Time system C1000 TM Thermal Cycler (Bio‐Rad), with the following cycling conditions: initial denaturation at 95°C (2 minutes), followed by 39 cycles of denaturation at 95°C (5 seconds), combined annealing and extension at 60°C (20 seconds). Gene expression was normalized to the geometric mean of selected reference genes shown in Table 1. Reference genes which exhibited stable expression levels after DEX and cortisol treatments in the first trimester explants were as following: the zeta polypeptide (*YWHAZ*) and succinate‐ubiquinone oxidoreductase (*SDHA*). The reference genes used following treatments in the Bewo cells were as follows: DNA topoisomerase 1 (*TOP1*) and 18S ribosomal RNA (*18S*).

**Table 1:** List of primers used in this study

Primer name	Sequence	Reference
*ABCB1* a	Forward: 5′‐GCC CTTGTTAGACAG CCT CA‐3′	
	Reverse: 5′‐GGC TTTGTC CAG GGCTTCTT‐3′	
*ABCG2*	Forward: 5′‐TGGAATCCAGAACAGAGCTGGGGT‐3′	
	Reverse: 5′‐AGAGTTCCACGGCTGAAACACTGC‐3′	19
*GR*	Forward: 5′‐TTTCAGCTAACATCTCGGG‐3′	
	Reverse: 5′‐CTATGCATGAAGTGGTTGAAAA‐3′	39
*SYN2*	Forward: 5′‐GAAGGCCCTTCATAACCAATGA‐3′	
	Reverse: 5′‐GATATTTGGCTAAGGAGGTGATGTC‐3′	40
*MKP1/DUSP1*	Forward: 5′‐CCTTCCTCCAGCATTCTTGA‐3′	
	Reverse: 5′‐CAGTACAAGAGCATCCCTGTG‐3′	41
*YWHAZ*	Forward: 5′‐ACTTTTGGTACATTGTGGCTTCAA‐3	
	Reverse: 5′‐CCGCCAGGACAAACCAGTAT‐3′	42
*TOP1*	Forward: 5′‐GATGAACCTGAAGATGATGGC‐3′	
	Reverse: 5′‐TCAGCATCATCCTCATCTCG‐3′	42
*SDHA*	Forward: 5′‐TGGGAACAAGAGGGCATCTG‐3′	
	Reverse: 5′‐CCACCACTGCATCAAATTCATG‐3′	42
*18S* a	Forward: 5′‐GTAACCCGTTGAACCCCAATT‐3′	
	Reverse: 5′‐CCATCCAATCGGTAGTAGCG‐3′	

GR, glucocorticoid receptor.

a Gene specific primers were designed with primer‐BLAST(http://www.ncbi.nlm.gov/tools/primer-blast).

### 2.7 Protein extraction and immunoblotting

Western blot analysis was conducted as previously described.^3^, ^5^ Briefly, protein isolated from placental explants and cultured cells were extracted by sonication using lysis buffer (1 mol/L Tris‐HCL pH 6.8, 2% SDS, 10% glycerol containing protease and phosphatase inhibitor cocktail; Thermo Scientific). Protein concentration was determined with the Pierce BCA Protein assay kit (Thermo Scientific). Proteins were separated by electrophoresis (30 μg 100V, 1 hour) using SDS polyacrylamide gels (7%) and then transferred (10 minutes) to polyvinylidene fluoride (PVDF) membrane using Transfer Pack Quick Start Guide (Bio‐Rad). Membranes were blocked with skim milk (5%; 1 hour) for all proteins except MDR1 (P‐gp), where 5% bovine serum albumin (BSA) in Tris‐buffered saline containing 0.1% Tween was used. The primary antibodies used were anti‐rabbit MDR1 (P‐gp, dilution 1:1000; Abcam), anti‐rabbit BCRP (dilution 1:3000; Abcam), anti‐rabbit HERV (dilution 1:100; Abcam), anti‐mouse CK7 (dilution 1:1000; Dako), anti‐rabbit ERK2 (dilution 1:2000; Santa Cruz Biotechnology) and anti‐rabbit ß‐actin (Bio Vision, Milpitas Blvd, Milpitas, CA). Blots were incubated with primary antibodies overnight at 4°C. The PVDF membranes were subsequently incubated for 1 hour with HRP‐linked anti‐rabbit secondary antibody (GE Healthcare Bio‐Science, Baie d'Urfe, QC, Canada) at concentrations of 1:10 000 for P‐gp, CK7, ERK2 and ß‐actin, and 1:15 000 for BCRP. Protein‐antibody complexes were detected by incubating (5 minutes) the PVDF membranes with Laminate Crescendo Western HRP Substrate (Millipore, Oak Drive, CA), and the chemiluminescence was detected under UV using ChemiDoc™ MP Imaging system (Bio‐Rad). The protein band intensity was quantified using Image Lab™ software and normalized against the ß‐actin signal.

### 2.8 Lactate dehydrogenase cytotoxic assay

Viability of the villous explants treated with DEX or cortisol (10^−8^, 10^−6 ^mol/L) was determined by measuring LDH leakage into the medium as previously described.^19^, ^23^ LDH was quantified with the Cytotoxicity Detection kit (Roche Applied Science) according to the manufacturer's instructions. A standard curve for the LDH assay was generated with LDH from rabbit muscle (Sigma‐Aldrich), and absorbance was measured at 490 nm (BioTek Instruments Inc., Winooski, VT). The LDH concentration in the media was normalized to placental explant weight.

### 2.9 Statistical analysis

Exploratory data analyses were performed with Prism version 6 (GraphPad Software Inc., San Diego, CA). Differences in explant protein levels were assessed by paired t tests in placental explants and by one‐way ANOVA followed by the Newman‐Keuls post hoc test in BeWo cells. Differences in mRNA levels in explants exposed to DEX and cortisol and culture medium LDH contents were assessed with two‐way ANOVA, followed by Bonferroni’ s test. Statistical differences were set at *P* < .05.

## 3 RESULTS

### 3.1 Glucocorticoids increase the expression of *ABCB1* and *ABCG2*/BCRP in first trimester ex vivo placental explants

**Figure 1:**
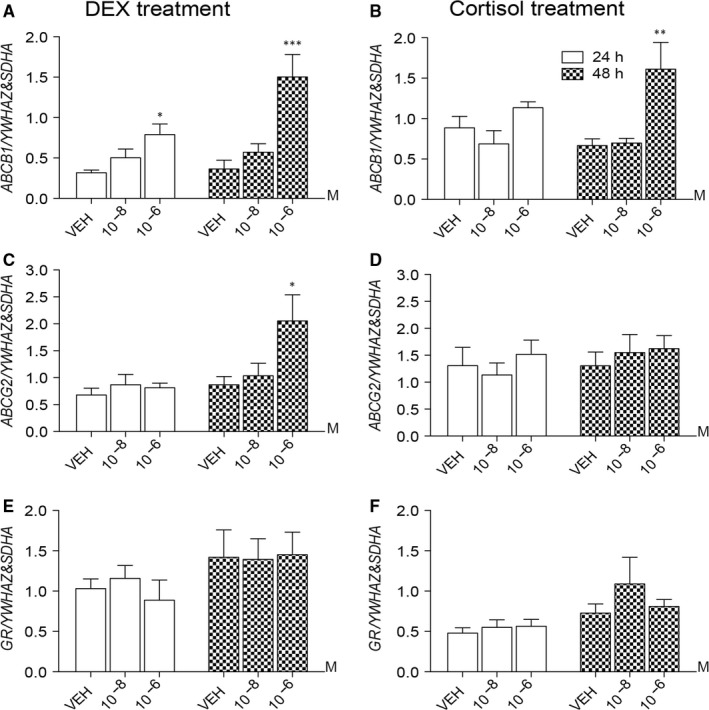
Effect of dexamethasone (DEX) and cortisol on *ABCB1, ABCG2* and glucocorticoid receptor (*GR*) *mRNA* levels in first trimester human placental villous explants. (A&B) *ABCB1*, (C&D) *ABCG2* and (E&F) *GR* mRNA expression (n = 8 placentae/group) in first trimester villous explants (7‐10 weeks) following treatments with DEX and cortisol, respectively; for 24 (open bars) or 48 hour (hatched bars). *P <* *.05, **P <* *.01 and ***P <* *.001 vs VEH. Data are presented as mean ± SEM

**Figure 2:**
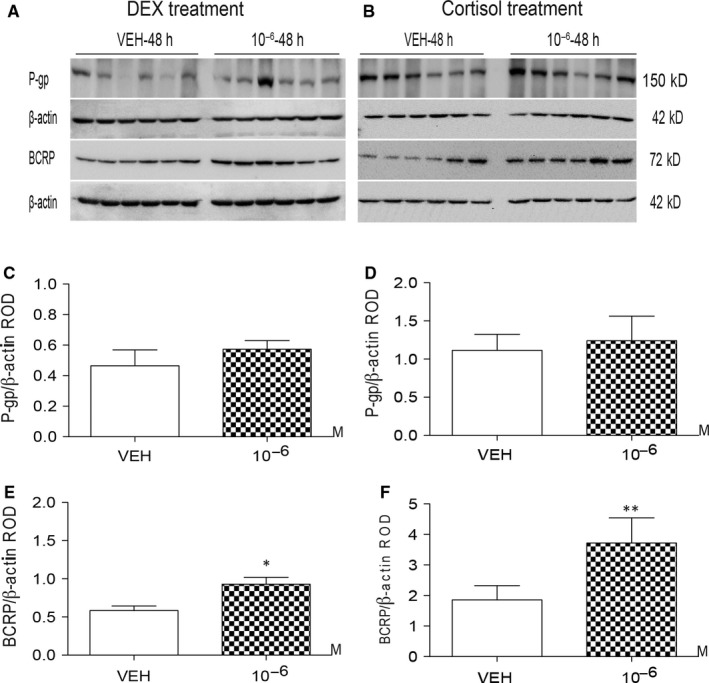
Effect of dexamethasone (DEX) and cortisol on P‐gp and breast cancer resistance protein (*BCRP*) protein levels in first trimester human placental villous explants. (A&B) Western blot analysis of P‐gp and BCRP levels in first trimester placental explants (n = 6 placentae/group) treated with DEX (A; 10^−6 ^mol/L), cortisol (B; 10^−6^ mol/L) or VEH for 48 hour. Corresponding mean P‐gp (C&D) or breast cancer resistance protein (BCRP) (E&F) expression relative to β‐actin *P <* *.05, **P <* *.01 vs VEH. Data are presented as mean ± SEM

DEX (10^−6^ mol/L) increased the levels of placental *ABCB1* mRNA at 24 hour and 48 hour post‐treatment (*P *<* *.05 and *P *<* *.001), and increased *ABCG2* mRNA levels at 48 hour (*P *<* *.05) (Figure 1A & C). Cortisol (10^−6^ mol/L) increased *ABCB1* expression (*P *<* *.01) after 48 hour (Figure 1B). The lower dose of DEX and cortisol (10^−8^ mol/L) did not affect placental *ABCB1* and *ABCG2* mRNA at either time‐point. Glucocorticoid receptor mRNA levels were not affected by either DEX or cortisol treatment (Figure 1E & F). Dexamethasone and cortisol treatments (10^−6^ mol/L) increased BCRP protein levels (*P *<* *.05 and *P *<* *.01) (Figure 2A,B,E & F); however, no changes in P‐gp protein levels were detected (Figure 2A,B,C & D). Lactate dehydrogenase toxicity assays showed that DEX and cortisol had no effect on the viability of placental explants in culture (data not shown).

### 3.2 Expression of BCRP is enriched in first trimester human syncytiotrophoblast cells

**Figure 3:**
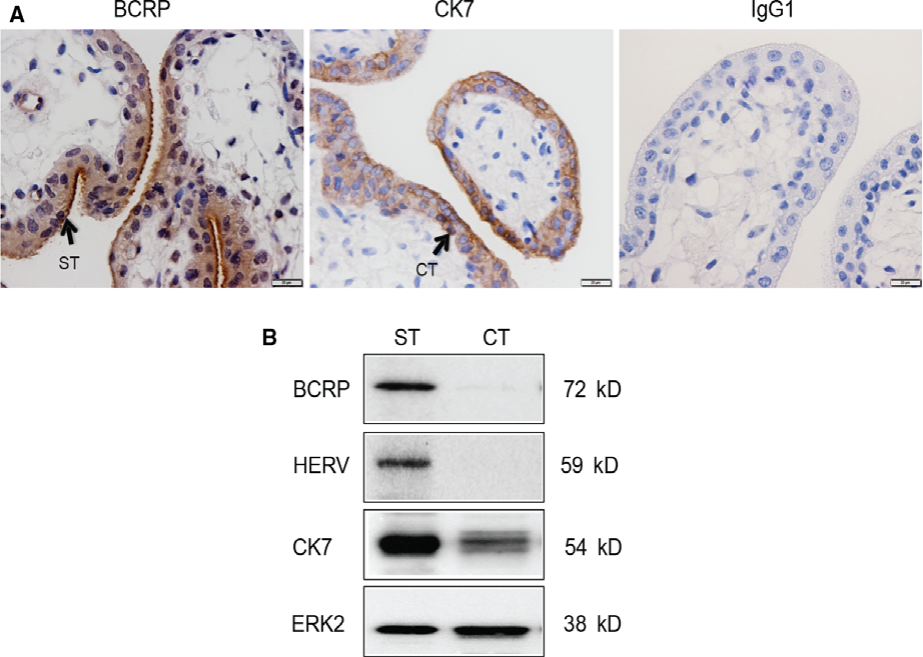
Breast cancer resistance protein localization and protein levels in first trimester human placenta (7‐10 weeks). (A) Representative immunostaining images of placental BCRP, cytokeratin 7 (CK7: a trophoblast marker) and IgG1 (control) in first trimester human placenta (n = 6). BCRP is highly localized at the apical surface of the syncytiotrophoblast (ST) and to a lesser extent at cytotrophoblast (CT) cells. (B) Representative Western blot analysis of BCRP, HERV (marker of syncytial trophoblasts), CK7 and ERK2 (loading control) levels in isolated first trimester CT and ST cells

As we observed glucocorticoid‐induced up‐regulation of placental BCRP expression, we undertook detailed analysis of BCRP localization in first trimester explants. Immunohistochemical analysis revealed BCRP immunostaining in the syncytiotrophoblasts, with high levels at the apical surface (Figure 3). BCRP immunostaining was present but at lower levels in the cytotrophoblast cells. This pattern is corroborated by the localization of CK7 (a specific marker for trophoblast tissue), which stained both cytotrophoblast and syncytiotrophoblast cells (Figure 3). Western blot analysis of isolated cytotrophoblast and syncytiotrophoblast cells from first trimester placentae revealed that BCRP protein levels were highest in the syncytiotrophoblast cells, although residual expression was detected in the isolated cytotrophoblast cells (Figure 3B). This pattern of expression suggests that first trimester syncytiotrophoblast cells are the site of BCRP up‐regulation in response to glucocorticoid treatments. HERV (a specific marker for syncytial trophoblasts) was only detected in syncytiotrophoblast cells, whereas CK7 was detected in both syncytiotrophoblast and cytotrophoblast cells, verifying the purity of isolates.

### 3.3 DEX increases BCRP expression in BeWo cells after syncytialization

**Figure 4:**
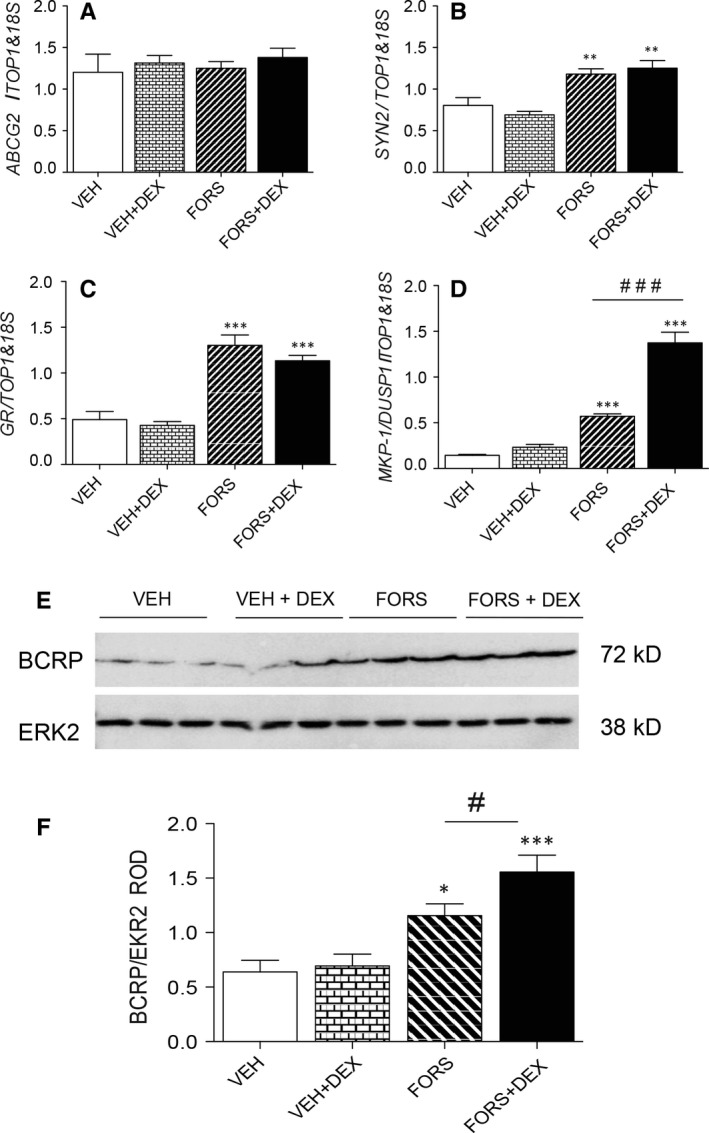
Effects of dexamethasone (DEX) on breast cancer resistance protein (*BCRP*) expression in non‐syncytialized and syncytialized BeWo cells. (A) *ABCG2*, (B) *SYN2,* (C) glucocorticoid receptor (*GR*) and (D) *MKP‐1/DUSP1* mRNA expression (n = 6 independent experiments) in non‐syncytialized and syncytialized (induced by 72‐hour forskolin treatment) BeWo cells treated with DEX (10^−6^ mol/L) or VEH for a further 72 hour. (E) Representative Western blot of BCRP protein following the various treatments and (F) corresponding quantification of BCRP protein levels relative to ERK2. *P < .05, **P <* *.01, ***P <* *.001 vs VEH; ^#^ *P < .05* represents differences between forskolin vs forskilin + DEX treatments. Data are presented as mean ± SEM

To better explore the hypothesis that syncytiotrophoblast cells are the site of glucocorticoid‐mediated BCRP up‐regulation in the placenta, the BeWo cell model was used to study the effect of DEX on BCRP expression in non‐syncytialized (cytotrophoblast phenotype) and syncytialized (syncytiotrophoblast phenotype) states (Figure 4). Syncytialization was induced with 25 μmol/L forskolin (72 hour), and cells were treated with DEX (10^−6^ mol/L) or VEH for an additional 72 hour. Forskolin treatment increased the expression of *Syn2* (a marker of syncytialization; *P *<* *.01; Figure 4B), *GR* mRNA (*P *<* *.001; Figure 4C) and *MPK1/DUSP1* (*P *<* *.001; Figure 4D), an established marker of glucocorticoid responsiveness^24^ but had no effect on *ABCG2* mRNA (Figure 4A). By contrast, forskolin treatment significantly increased BCRP protein levels (*P* < .05), and the addition of DEX, further augmented BCRP protein expression (*P* < .05; Figure 4F). Dexamethasone exposure after forskolin treatment further increased the expression of *MPK1/DUSP1* (*P *<* *.001; Figure 4D), but had no additional effect on *SYN2* and *GR* mRNA, and had no effect on *ABCG2* mRNA.

## 4 DISCUSSION

This study is the first to show that glucocorticoids increase the expression of *ABCB1* mRNA, *ABCG2* mRNA and BCRP protein in the first trimester human placenta. Furthermore, we also demonstrated that BCRP is most highly expressed in the syncytiotrophoblast cells and that BCRP is up‐regulated following syncytialization of trophoblast cells. Of importance, this up‐regulation is further enhanced following exposure to DEX, suggesting that syncytiotrophoblast cells are the response site for glucocorticoid‐mediated BCRP up‐regulation in the placenta. Another important finding is that the mRNA expression of *GR* and *MPK1/DUSP1* (a marker of glucocorticoid responsiveness)^24^ is elevated following forskolin treatment of BeWo cells. This suggests that glucocorticoid‐responsive intracellular machinery becomes activated as cytotrophoblast cells undergo syncytialization and is consistent with our previous findings demonstrating that DEX promotes syncytiotrophoblast differentiation and maturation of human trophoblast explants.^25^

With regard to glucocorticoid modulation of *ABCB1* and P‐gp in the first trimester placenta, different time‐dependent effects of DEX and cortisol on *ABCB1* expression were observed. Glucocorticoid‐induced *ABCB1* expression has been previously demonstrated in animal models. Administration of high‐dose DEX to pregnant mice (from E12.5‐E18.5) increased placental *Abcb1a* mRNA and P‐gp protein on E18.5 (term).^26^ Further, DEX treatment of human trophoblast cell lines (JEG3 and BeWo) and human primary trophoblast cell cultures (derived from term placentae) also increased *ABCB1* mRNA expression.^27^, ^28^ Together, these results show consistent effects of glucocorticoids on placental *ABCB1* mRNA at different stages of pregnancy. However, we did not find changes in P‐gp protein expression after glucocorticoid exposure, in the human first trimester placenta, suggesting that effects of glucocorticoids on placental P‐gp expression depend upon treatment duration, species and/or gestational age. Furthermore, the disconnect between *ABCB1* mRNA and P‐gp protein levels following glucocorticoid treatment might be explained by microRNA regulation of mRNA transcripts of ABC transporters and subsequent translation. We have previously demonstrated that chorioamnionitis induces *ABCB1* mRNA expression but decreases P‐gp protein levels. In this context, we have identified that miR‐331‐5p, involved in P‐gp suppression, was concomitantly increased in chorioamnionitis and might explain the mismatch between mRNA levels and protein found in chorioamnionitis.^29^ It is possible that glucocorticoids regulate expression of microRNAs involved in *ABCB1* translation in the first trimester placenta. This possibility clearly warrants further investigation.

Placental *ABCB1* and P‐gp expression has been shown to be modulated by different factors. We have shown that *ABCB1* mRNA and P‐gp protein expression in first and third trimester human placental explants is altered by oxygen tension, lipopolysaccharide (LPS, bacterial antigen) and polyinosinic‐polycytidylic acid (poly[I:C], viral antigen).^3^, ^19^ Importantly, pregnant mice (E15.5‐mid to late pregnancy) exposed to LPS exhibited decreased placental P‐gp activity.^30^ In contrast, polyI:C did not alter P‐gp activity,^31^ indicating that infective stimuli alter P‐gp function in an insult‐specific manner. As other factors have been shown to modulate placental P‐gp in earlier and later stages of pregnancy, future studies should investigate how glucocorticoids impact the placental expression of *ABCB1* mRNA and P‐gp protein in the third trimester placenta.

We also found that glucocorticoids increased *ABCG2* mRNA and BCRP protein levels in first trimester placental explants. Dexamethasone treatment increased *ABCG2* expression 48 hour after exposure, whereas cortisol did not alter *ABCG2* levels at any dose or time‐points investigated. This indicates that natural and sGC affect placental *ABCG2* expression differently, which may be because of the differences in their downstream signalling mediators as DEX activates GR alone while cortisol simultaneously binds to both the GR and MR.^11^, ^12^ Moreover, DEX is a long acting glucocorticoid, considered to have 25‐ to 50‐fold greater potency than the short acting cortisol.^32^ Thus, the activation of different intracellular pathways by DEX and cortisol, relative potency and duration of action may underline the specific DEX up‐regulation of *ABCG2* mRNA levels found in the present study. Nonetheless, both DEX and cortisol up‐regulated BCRP after 48‐hour treatments, showing that glucocorticoids can regulate BCRP in the first trimester placenta. Glucocorticoid‐induced increase in placental BCRP expression has the potential to decrease exposure of the developing embryo or foetus to endogenous and exogenous BCRP substrates, such as folate, antibiotics and antiretrovirals.^5^ In sharp contrast, pregnant mice chronically exposed to high‐dose DEX later in gestation (on E15.5), exhibited decreased placental *Abcg2* mRNA and BCRP function.^15^ Importantly, this effect was more evident in female foetuses and was not evident on E18.5 (term). Differences in the number of DEX treatments, timing of exposure and model species may account for the different placental BCRP responses to glucocorticoids in these studies. Importantly, DEX has also been shown to increase expression of other placental transporters, including the amino acid transport system A, and to promote syncytiotrophoblast differentiation and maturation at term.^25^, ^33^, ^34^ One limitation of the present study is our inability to effectively assess of P‐gp and BCRP function in placental explants; an efficient assay is not currently available.

Using immunohistochemistry, we and others have reported that BCRP is enriched in the syncytiotrophoblast, while there is lower expression in cytotrophoblast cells.^3^, ^5^, ^35^ In the present study, we replicated these previous findings. By isolating cytotrophoblast and syncytiotrophoblast cells from first trimester placentae, we further confirmed that BCRP expression is enriched in syncytiotrophoblast cells. Residual expression of BCRP was also evident in isolated cytotrophoblast cells, which is consistent with our current and previous immunohistochemical studies.^20^

As we observed increased placental BCRP expression following DEX treatment, which promotes syncytiotrophoblast differentiation and maturation in vitro,^4^, ^25^ we set out to investigate whether cytotrophoblasts, or syncytiotrophoblasts, both of which express GR,^36^ are responsible for promoting BCRP up‐regulation following glucocorticoid exposure. We utilized the *in vitro* cytotrophoblast fusogenic model “BeWo cell,” which undergoes cell fusion and syncytialization following forskolin exposure.^37^ Induction of BCRP expression was observed upon syncytialization with forskolin. In this context, human primary cytotrophoblast cells isolated from term pregnancies increased *ABCG2* and BCRP expression as they syncytialize.^38^ This suggests that the BCRP‐mediated foetal protective barrier forms upon cytotrophoblast fusion and syncytialization. Furthermore, here we show that DEX enhances BCRP expression following syncytialization, suggesting that glucocorticoids may represent an important inducer of development and differentiation of the trophoblast protective barrier throughout pregnancy, or at least, during the first trimester of gestation, a hypothesis that clearly requires further investigation.

We conclude that glucocorticoids exert selective effects on expression of key multidrug transporters in the human first trimester placenta. Specific effects depend on the type of glucocorticoid, dosage and length of exposure. Breast cancer resistance protein protein is highly expressed in the syncytiotrophoblasts, demonstrating that it is this trophoblast layer that provides BCRP‐mediated protective barrier. Exposure of first trimester placental cells to glucocorticoids increases the expression of BCRP in syncytializing and/or syncytialized trophoblast cells, which enhances the placenta barrier to toxins, drugs and endogenous substrates. This suggests that maternal stress and or sGC administration during early stages of pregnancy has the potential to alter placental barrier permeability to a number of BCRP substrates present the maternal circulation. Our data offer evidence that syncytiotrophoblasts are already responsive to glucocorticoids at early stages of pregnancy and that glucocorticoids induce a further increase in the expression of drug transporter proteins and provide control over the factors entering the foetal compartment.

## CONFLICTS OF INTEREST

The authors report no conflicts of interest in this work.
